# Relationship between clonogenic radiosensitivity, radiation-induced apoptosis and DNA damage/repair in human colon cancer cells

**DOI:** 10.1038/sj.bjc.6601427

**Published:** 2003-12-09

**Authors:** A L Dunne, M E Price, C Mothersill, S R McKeown, T Robson, D G Hirst

**Affiliations:** 1 School of Biomedical Sciences, University of Ulster, Newtownabbey, BT37 OQB, Northern Ireland; 2 Radiation Science Centre, Dublin Institute of Technology, Dublin

**Keywords:** DNA strand breaks, predictive assay, apoptosis, colon cancer

## Abstract

The intrinsic radiation sensitivity of normal and tumour tissue is a major determinant of the outcome of radiotherapy. There is currently no established test that can be used routinely to measure the radiosensitivity of the cells in an individual patient's cancer in a manner that can inform treatment planning. The purpose of this study was to evaluate, in four human colorectal adenocarcinoma cell lines, two possible end points as surrogate markers of radiation response – apoptosis and induction of DNA single-strand breaks – and to compare the results with those of a conventional clonogenic assay. Cell lines (SW707 SW480, SW48 and HT29) known to differ in radiosensitivity were exposed to single doses of X-rays ranging from 0.5 to 5 Gy and cell survival was measured using the clonogenic assay. Apoptosis was determined on the basis of morphology under fluorescent microscopy and DNA damage/repair was measured, as tail moment, using an adaptation of the alkaline comet assay. The relationship between surviving fraction at 2 Gy (SF_2_) and the percentage of apoptotic cells 24 h after the same dose was complex, but apoptosis accurately predicted the order of radiosensitivities as measured by SF_2_. Initial damage measured after 2 Gy using the alkaline comet assay gave a close correlation with SF_2_ (*r*^2^=0.95), whereas there was no correlation between initial DNA damage repair rate and SF_2_.

Cellular radiosensitivity has long been a research focus in the field of radiation biology and oncology because it has a clear influence on the outcome of therapy (Hu and Hill, 1996). Direct evidence that the intrinsic radiosensitivity of tumour cells is an important determinant of patient response to radiotherapy has been gained from experiments using a soft agar clonogenic assay (West, 1995, 1997). While this technique has provided useful data, it has the important disadvantage that a minimum of 4 weeks is required for colony growth, and success rates in obtaining survival fraction at 2 Gy (SF_2_) values are only around 70%, even in the most experienced laboratories (Davidson and West, 1990; West, 1992). The limitations of this clonogenic method highlight the need for development of new rapid, predictive assays of radiation responses. If the radiosensitivity of tumours could be predicted, it may eventually allow the individualisation of patient treatment by radiotherapy (Wilks *et al*, 1996).

The obvious candidate marker is the double-strand break. Its relationship to clonogenic cell survival has been studied by several investigators and correlations were found in some studies (Radford, 1986; Prise *et al*, 1987) though not all (McMillan *et al*, 1990). A weakness of this approach has been that the dose range of the end points does not overlap; to generate a measurable number of double-strand breaks, much higher radiation doses are required than are appropriate for the clonogenic assay or in the clinical setting. However, interest in the double-strand break, as a practical end point has recently been enhanced by the development of a very different approach to the measurement of radiation-induced DNA damage (Rothkam and Lobrich, 2003; MacPhail *et al*, 2003). This exploits phosphorylation of histone H2AX at the sites of double-strand breaks and has the advantage of detecting damage after doses as low as a few cGy (MacPhail *et al*, 2003).

Micronucleus and apoptosis assays have been used and studied widely as biological indicators for cellular radiosensitivity; however, there is no clear agreement regarding the usefulness of these methods. Several authors have reported a good quantitative inverse relationship between micronucleus frequency and clonogenic survival (Wandl *et al*, 1989; Jamali and Trott, 1996; Mariya *et al*, 1997); however, there are also several reports in which such a correlation was not found (Bush and McMillan, 1993; Slavotinek *et al*, 1993; Villa *et al*, 1994). Some authors have reported an inverse correlation between apoptotic frequency and cell survival (Radford and Murphy, 1994; Olive and Durand, 1997; Guo *et al*, 1999), whereas others found no correlation (Yangihara *et al*, 1995; McKenna *et al*, 1996).

Several investigators have used the alkaline and neutral comet assays to examine the possible relationship between radiosensitivity and DNA damage. The comet assay is a relatively easy method for detecting DNA damage and the results can be obtained within 24 h. In one study (McKelvey-Martin *et al*, 1998), the relationship between cell survival and induction of DNA single-strand breaks was assessed in three bladder transitional cell carcinoma cell lines using the alkaline comet assay; an inverse correlation was obtained between cell survival (clonogenic assay) and mean tail moment (comet assay) suggesting that the comet assay could be used to predict the radioresponsiveness of individual cell lines. It is still unclear, however, if there is a direct or quantitative relationship between DNA damage, apoptosis and radiosensitivity.

To date there have been no reports of a test that can be used routinely to predict the response of individual colorectal tumours to radiotherapy. The aim of the present study was to determine if either scoring of apoptotic cells, or measurement of DNA damage, using the alkaline comet assay, could have potential for use as a surrogate marker in determining the radiosensitivity of colorectal cancer cells.

## MATERIALS AND METHODS

### Cell lines and culture

Human colonic adenocarcinoma-derived cell lines SW707, SW48 and SW480 were kindly supplied by Dr R Guttenberger, Department of Radiotherapy, University of Freiburg, Germany. They were maintained in Leibovitzs L-15 medium containing 10% foetal calf serum (FCS), 1% sodium pyruvate, 1% penicillin/streptomycin and Hepes. The HT-29 cell line was cultured in McCoy's 5A-modified medium supplemented with 10% FCS, 1% penicillin/streptomycin and 1% L-glutamine. All cell lines were kept in an incubator at 37°C in humidified 5% CO_2_ and passaged by harvesting with trypsin-EDTA and seeding before they reached confluence. SW48 cells are wild–type for p53, whereas SW707, SW480 and HT29 cells are mutant.

### Clonogenic assay

Cells were subcultured into tissue culture flasks, then left in the incubator for 6 h to attach. The number of cells per flask was varied so that 100–200 colonies would survive after each of the different treatments. After 6 h, cells were irradiated at room temperature with doses of 0–5 Gy at a dose rate of 2.6 Gy/min^−1^ using a 300 kV X-ray machine (Siemans Stabilipan).

After irradiation, flasks were incubated at 37°C in a humidified atmosphere of 95% air/5% CO_2_ for 2 weeks to allow the formation of macroscopic colonies. The cells were then fixed with acetone and stained with crystal violet. Colonies containing >50 cells were counted and surviving fractions calculated after correction for plating efficiency of control cells. At least three independent experiments, each using duplicate flasks, were performed for each cell line.

### Microscopy and detection of apoptosis

For the studies of cell morphology, control and irradiated cells were harvested from the flasks at each time point using trypsin-EDTA and were prepared for cytocentrifugation. Cytospin preparations were made for each cell line. Thus, both attached cells and those that had become detached after irradiation were included in the scoring. Following fixation, the cells were stained with Diff Quick (Sigma) for light microscopy analysis and with DAPI (4′,6-diamidine-2′-phenylindole dihydrochloride) (Sigma) for fluorescent microscopy analysis. The extent of apoptosis was determined by examining the cells at × 20, × 40 and × 100 magnification. The cells were scored by their morphological characteristics as either normal or apoptotic. The morphological features used to classify cells as apoptotic were blebbing of the membrane, chromatin condensation and DNA fragmentation. A minimum of five fields of 100 cells were counted per experiment. Three independent experiments were performed at each time point and the data are given as the mean±s.e.

### Alkaline comet assay (slide method)

The alkaline comet assay was performed after irradiation of cells embedded in agarose gel, using an improved protocol (McKelvey-Martin *et al*, 1998) based on that first reported by Singh *et al* (1988). Preheated fully frosted microscope slides (Dakin) were covered with 95 *μ*l of 0.6% normal melting point agar (dissolved in RPMI medium and held at 45°C), coverslips were added and the agarose was allowed to solidify. Equal volumes of cell suspension/1.2% low melting point agar (dissolved in RPMI medium containing 10% FCS and held at 37°C) were mixed. The coverslips were removed and 75 *μ*l of this second layer was quickly pipetted onto a slide and allowed to solidify for 5 min under a fresh coverslip. The coverslips were then removed and the slides placed on ice and irradiated (0–5 Gy) as described in section Cell lines and culture. The slides were then immediately immersed in cold lysing solution (2.5 M NaCL, 100 mM NA_2_ EDTA, 10 mM Tris, pH 10 and 1% freshly added Triton X-100).

After 1 h in lysis buffer, slides were placed in a horizontal gel electrophoresis unit filled with fresh, chilled electrophoresis buffer (300 mM NaOH and 1 mM Na_2_ EDTA, pH 13.0) to a level of approximately 0.25 cm above the slides. To allow the unwinding of the DNA, slides remained in contact with the high pH buffer for 20 min. Electrophoresis was then carried out for 20 min at 25 V (0.83 V/cm^−1^). Slides were then drained, placed on a tray and flooded slowly with three changes of neutralisation buffer (0.4 Tris pH 7.5) each for 5 min, to remove alkali and detergents. Slides were then each stained with 50 *μ*l of ethidium bromide (20 *μ*g ml^−1^) and covered with a coverslip for immediate analysis. All the steps described were conducted under yellow light to prevent additional DNA damage by natural light.

A total of 100 cells per slide were scored for each of two replicate slides per experiment at each dose point by image analysis (Hewlett Packard Super VGA and Fenestra Komet Software, version 3; Kinetic Imaging Ltd). Observations were made at a magnification of × 400 using an epiflourescence microscope (Olympus BH2) equipped with an excitation filter of 515–535 nm, 100 W mercury lamp and a barrier filter at 590 nm. Several measures of DNA migration were calculated by the software package for each cell, but tail moment was selected as the parameter that best reflected DNA damage. This is defined as the tail length multiplied by the percent tail DNA, where tail length is defined as comet length minus head length.

### Data analysis

All experiments were repeated three times and two replicates were performed for each point within each independent experiment. Statistical analysis was performed using the Student's *t*-test and analysis of variance.

## RESULTS

### Sensitivity of colorectal tumour cells to X-rays determined by clonogenic assay

**Figure 1:**
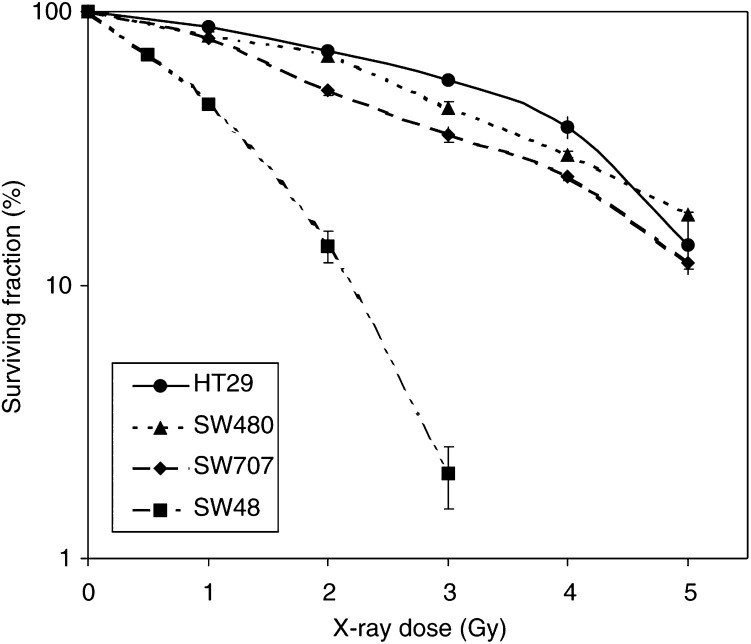
Radiation cell survival curves for four colon adenocarcinoma cell lines. Error bars represent ±1 s.e.m.

Radiation survival curves for the four colorectal cancer cell lines are shown in Figure 1

, with the SF_2_ value (fraction of cells surviving exposure to 2 Gy) for each cell line given in Table 1

**Table 1:** Comparison of clonogenic cell survival of four colon cancer cell lines with initial DNA damage and initial DNA repair rate assessed using the comet assay

	**Clonogenic assay**	**Comet assay (initial damage)^a^**	**Comet assay (damage rejoining)^b^**		
Cell Line	SF_2_±1 s.e.m.^c^	Tail moment U Gy^−1^±1 s.e.m.	d[r^2^]	Initial (0–15 min) repair half time after 5 Gy (min)	[r^2^]^d^
HT29	0.72±0.29	2.02±0.13	0.992	13.7	0.83
SW480	0.69±0.32	2.20±0.08	0.997	10.7	0.79
SW707	0.50±0.19	2.64±0.13	0.993	12.2	0.90
SW48	0.14±0.18	3.99±0.10	0.999	13.7	0.87

a Initial damage per unit dose expressed as the linearly fitted slope of the four observations (0–5 Gy) for each dose–response curve from Figure 3.

b Rate of damage rejoining expressed as the slope of the exponential fit to the first three observations (0–15 min) for each curve from Figure 4.

c Fraction of cells surviving exposure to 2 Gy.

d Correlation coefficient of fit.

. There was a wide variation in the SF_2_ values obtained, with the SW48 cell line showing much greater sensitivity than any of the other cell lines.

### Radiation-induced apoptosis

**Figure 2:**
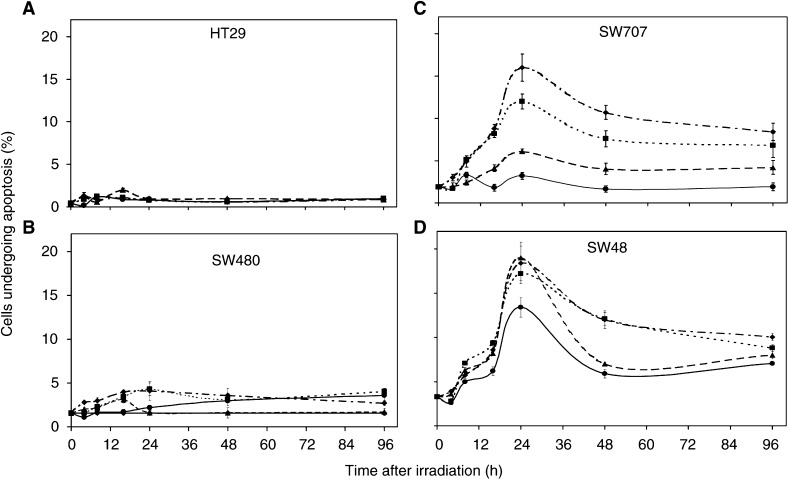
Apoptotic frequency for four colon adenocarcinoma cell lines at different times after a range of X-ray doses: 0.5 Gy (•), 1.0 Gy (▴), 2.0 Gy (▪) and 5 Gy (♦). Error bars represent±1 s.e.m.

The colon cancer cell lines were assessed for apoptosis at 4, 8, 16, 24, 48 and 96 h post-irradiation (0–5 Gy X-rays). The percentage of apoptotic cells for each of the four cell lines at different times after 0.5, 1, 2 or 5 Gy is shown in Figure 2A–D

. Apoptosis was seen in all control cultures though they represented only 0.3–4% of the cells observed, depending on the cell line. The percentage of apoptotic cells increased significantly with increasing X-ray dose in both SW48 and SW707 cultures (*P*<0.0001). There was a small but significant (*P*<0.05) increase in apoptosis in SW480 cells and no increase in HT-29 cells. Apoptosis increased with time after irradiation and reached a maximum at 24 h for SW707, SW48 and SW480 cell lines and then declined gradually over the next 3 days. This suggests that the most appropriate time to conduct an apoptosis assay on colon cancer cells is 24 h after irradiation, as the value at that time should represent the maximum.

### Radiation dose–response curves for DNA damage as measured by the comet assay

**Figure 3:**
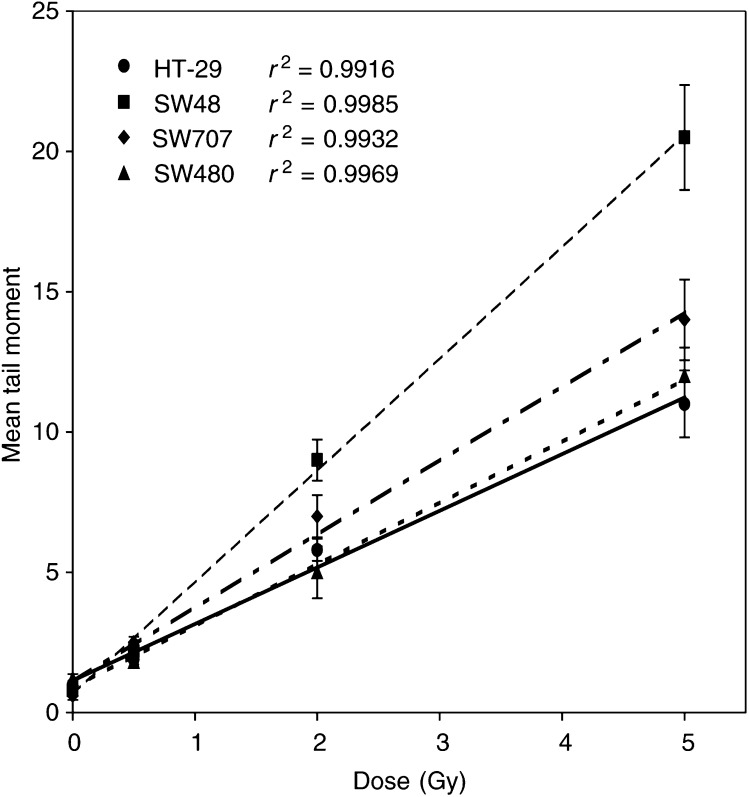
DNA damage measured immediately after irradiation by the alkaline comet assay, expressed as mean tail moment as a function of radiation dose (0.5, 2 and 5 Gy) in four colon adenocarcinoma cell lines. Error bars represent ±1 s.e.m.

The dose–response curves generated for the colorectal cancer cell lines by assaying for DNA damage immediately after irradiation on ice are shown in Figure 3

. Linear regression analysis revealed that the relationship between dose and tail moment was linear, over the 0–5 Gy dose range tested, for each of the cell lines, although the amount of DNA damage per unit dose (Table 1) varied by two-fold between the most radioresistant (HT29) and the most radiosensitive (SW48) cell lines; this difference was highly significant (*P*<0.0001).

### Comparison of DNA repair rates after irradiation

**Figure 4:**
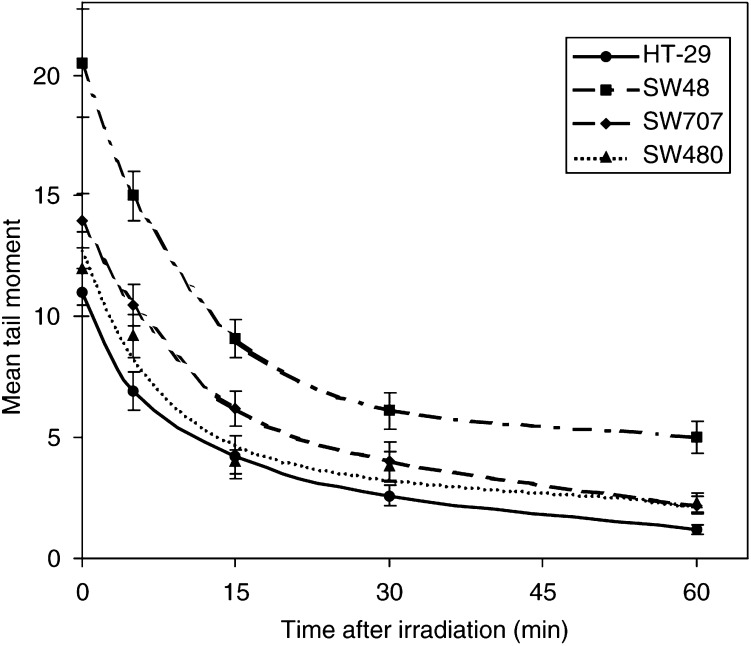
DNA damage expressed as mean tail moment, measured by the alkaline comet assay, as a function of time after a radiation dose of 5 Gy in four colon adenocarcinoma cell lines. Error bars represent±1 s.e.m.

The DNA repair characteristics (repair of single-strand breaks) of the four cell lines were examined by allowing the cells to incubate at 37°C for various times after 5 Gy before subjecting the cells to comet analysis. The repair curves are shown in Figure 4

. The values at time zero are those also shown for 5 Gy in Figure 3, illustrating clear differences in ‘initial’ damage. The kinetics of repair of damage for all cell lines were complex and were not well fitted by an exponential or second-order polynomial. However, the first three time points (0–15 min) were a good fit to an exponential and the repair half times are shown in Table 1. There is no significant difference (*P*>0.05) between any of the repair half times.

### Correlation between initial mean tail moment and SF_2_, and repair rate and SF_2_

**Figure 5:**
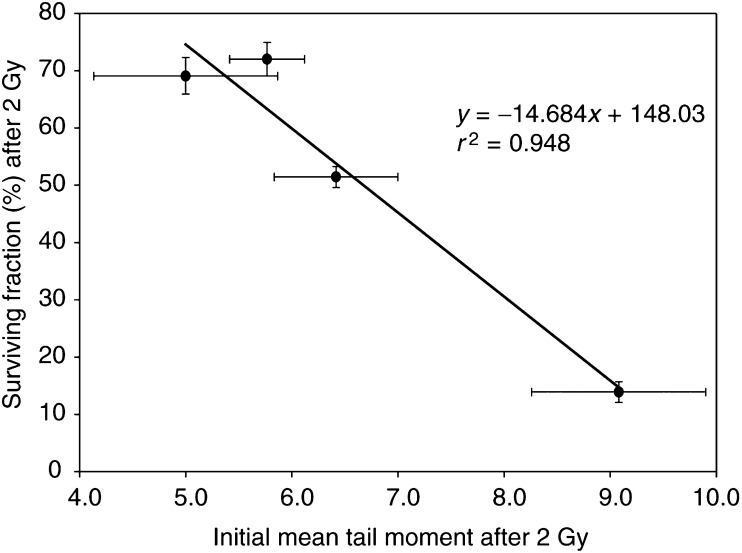
Correlation between surviving fraction after 2 Gy X-rays and DNA damage expressed as mean tail moment, measured immediately after 2 Gy by the alkaline comet assay. Error bars represent±1 s.e.m.

The relationship between initial mean tail moment and SF_2_ is plotted in Figure 5

. There is a strong and correlation and where cell lines had almost identical SF_2_ values, initial mean tail moment was not significantly different. By contrast, initial repair rates (Table 1) were very similar in all cell lines.

### Correlation between apoptosis frequency and SF_2_

**Figure 6:**
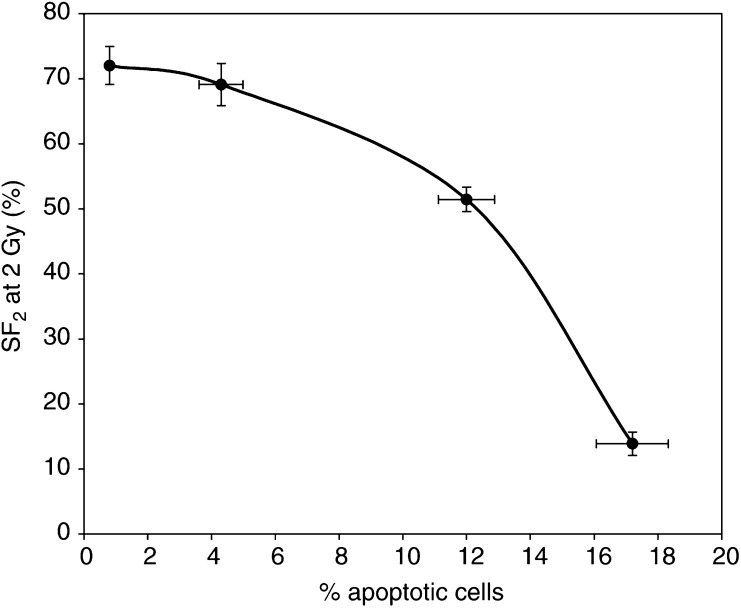
Relationship between surviving fraction after 2 Gy X-rays and the percentage of apoptotic cells. Error bars represent ±1 s.e.m.

The correlation between apoptotic frequency at 24 h and SF_2_ is shown in Figure 6

. The end points were clearly related such that the increasing frequency corresponded with reduced SF_2_, and apoptosis frequency predicted the correct order of radiosensitivities of the four cell lines, but the data did not fit any simple mathematical relationship.

## DISCUSSION

Predictive assays of tumour radiosensitivity would make an important contribution to enhancing the effectiveness of radiotherapy, if they could provide timely information allowing treatments to be planned specifically for the individual patient (Guo *et al*, 1999). There have been a number of surveys of the variability in radiosensitivity between and within various classes of mammalian cells as well as of the potential mechanisms for such sensitivity differences. In the present study, we observed a five-fold difference in radiosensitivity (measured by SF_2_) between the most sensitive (SW48) and the most resistant (HT29) colon tumour cell lines. This is consistent with other published data (Leith *et al*, 1991). If fully reflected *in vivo*, this difference would profoundly affect treatment outcome. Determination of SF_2_ by clonogenic assay, however, takes 20 days to complete, so there is a need for the development of more rapid methods of measuring cellular radiosensitivity in time to inform individual treatment planning.

It has been proposed that differences in the patterns of apoptotic death could be an explanation for differences in radiosensitivity (Olive and Durand, 1997), particularly if cell lines capable of undergoing rapid apoptosis tolerate less DNA damage. A study by Dewey *et al*. (1995) compared the fraction of cells that underwent apoptosis shortly after irradiation with the clonogenic survival of the population. They concluded that apoptosis measured within 24 h after irradiation could account for only a fraction of the total clonogenic cell kill, and consequently that mitotic death must be responsible for a significant proportion of the observed cytotoxicity. Several studies suggest that an enhanced apoptotic response results in greater sensitivity (Story *et al*, 1992; Russell *et al*, 1995); however, there are also reports showing that interference with apoptosis does not affect radiosensitivity (Agaki *et al*, 1993). One possible explanation for this is that there may be a poor correlation between apoptotic cell frequency of attached cells and clonogenic cell survival (Guo *et al*, 1999). These results indicated that the occurrence of apoptosis among attached cells following irradiation depended on the cell type, and that apoptosis among attached cells could not predict the radiosensitivity of cell lines. Indeed, floating cells have been shown to account for most of the apoptosis *in vitro* (Darzynkiewicz *et al*, 1997), probably because the process of apoptosis tends to cause detachment from the substrate (Dewey *et al*, 1995; Olive *et al*, 1996). The colorectal cancer cells used in the present study remained floating in the medium for up to 48 h. For this reason we scored apoptosis in all cells, both floating and attached.

The percentage of cells undergoing apoptosis at any given time differed markedly between the four cell lines (Figure 2). The background apoptosis frequency was very low (<1%) in the HT29 cell line and there was no significant effect of radiation; in the SW480 cells, the background level was higher (2%) and increased significantly (*P*<0.05) at 24 and 48 h after 2 and 5 Gy; the background level in SW707 cells was again about 2%, but it increased in a dose-dependent manner to a well-defined maximum by 24 h after irradiation; similar results were obtained for SW48 cells though the dose–response relationship was not clearly defined, such that even a low dose of 0.5 Gy induced the same level of apoptosis as 5 Gy at 24 h. It is of interest that SW48 is the only cell line in our study expressing wild–type p53; considerable apoptosis was also seen in SW707 (mutant p53 and moderately radiosensitive) at 2 Gy, suggesting that p53 status alone is not a robust marker for radiosensitivity in colorectal cancer, a conclusion that is consistent with at least one clinical study of rectal cancer (Nehls *et al*, 1999). Apoptotic frequency peaked at 24 h for all the cells, but at that time the relationship to SF_2_ could not be defined in simple mathematical terms. Many more cell lines would need to be investigated before this could be achieved.

The comet assay is attractive as a potential clinical test as it requires few cells and the results can be available in a few hours. McKelvey-Martin *et al* (1998) assessed the ability of the alkaline comet assay to predict the radioresponsiveness of three bladder tumour cell lines. Well-defined radiation dose–response curves were observed with the greatest DNA damage displayed by the radiosensitive cell line and the least by the radioresistant cell line. McKeown *et al* (2003) and Moneef *et al* (2003) have shown that initial damage and residual damage correlate strongly with the radiosensitivity of bladder cancer cell lines, although the repair rate was not a robust marker. We now report similar results using four cell lines derived from colorectal cancers. Both initial damage and residual damage (at 60 min) measured using the alkaline comet assay correlate strongly with SF_2_, whereas repair half times were very similar for each cell line (identical for the most resistant and the most sensitive) and hence did not predict for clonogenic cell survival after irradiation. Residual damage measurement has the advantage that it should incorporate any differences in mismatch or nuclear excision repair that would contribute to the outcome; however, it is more difficult to measure accurately, and at least for the cell lines in the present study, significant differences in repair rates were not seen. A case could therefore be made for selecting the quickest and simplest end point for clinical application: initial damage. The concept of initial damage requires some discussion, however. The experimental protocol for the slide method of the alkaline comet assay requires that all procedures during and after irradiation, including cell lysis, are carried out at 0°C; therefore, it has been widely assumed that repair of DNA single-strand breaks is impossible. There is evidence to suggest, however, that this assumption may need to be considered more closely. While there is no evidence for repair of X-ray-induced single-strand breaks at 0°C, efficient repair of UV-induced breaks in V79 cells at 4°C has been reported (Bock *et al*, 1998). In contrast, more complex chormosomal lesions in human lymphocytes were not repaired at all at temperatures below 17°C (Virsik-Peuckert and Harder, 1986). Therefore, we should probably not entirely rule out the possibility of some repair process contributing to the value for ‘initial’ damage seen in the present study though it remains unlikely. Even if such a mechanism did occur, the kinetics would have to be very different in the four cell lines to invalidate the conclusion that the initial level of damage is different. This seems improbable as the repair kinetics at 37°C are very similar in the four cell lines (Figure 4; Table 1).

The alkaline comet assay does not predominantly measure the lesions (double-strand breaks) that are believed to be the cause of cell mortality – although a small component of the DNA breaks would have been double – rather it measures mainly single-strand breaks and alkali-labile sites. The credibility of any clinical assay of radiation sensitivity would be greatly enhanced if the biological relationship between the end point measured and cell death could be fully explained. This is probably an impossibly stringent requirement, although assays that measure double-strand breaks probably come close. The majority of these assays do, however, have one major disadvantage; they require radiation doses that are at least 10-fold higher than would ever be given in the clinic as single fractions, and any extrapolation to 2 Gy would have to rely on tumour cells in different individuals having approximately the same shaped survival curve. This is very unlikely to be the case. An exception to this is the recently developed method for detecting phosphorylated histone H2AX as a surrogate marker for the double-strand break (Rothkam and Lobrich, 2003; MacPhail *et al*, 2003). This exquisitely sensitive technique does not, however, have the ability to measure immediate damage as time must be allowed for the enzymic formation of gamma H2AX (MacPhail *et al*., 2003), during which time the DNA repair enzymes will be active. Furthermore, formation and loss of gamma H2AX is cell line dependent, so further studies will be needed to characterise this assay fully. Meanwhile, we believe that the alkaline comet assay has potential for use as a predictive test for colon and bladder cancer radiosensitivity. There are now encouraging data demonstrating the feasibility of using this technique in human tissues: a method for disaggregating colorectal tumour cells without compromising yield has been recently developed and has been used to investigate replicative integrity and DNA damage using a modification of the comet assay (McGlynn *et al*, 2003).

Ultimately, the robustness of the alkaline comet assay as a predictive test can only be fully established by testing on a much wider range of cell lines and human tumour tissues.
